# Modulation of mitochondrial DNA copy number: therapeutic potential of phytochemicals and plant extracts—a comprehensive review

**DOI:** 10.1007/s12272-026-01620-1

**Published:** 2026-05-10

**Authors:** Maria Rosaria Perri, Annamaria Cerantonio, Maria Grazia Cipriani, Ida Manna, Anna Aureli, Beatrice Marziani, Francesco Andreozzi, Gaia Chiara Mannino, Elettra Mancuso, Carolina Averta, Giancarlo Statti, Luigi Citrigno, Davide Mainieri

**Affiliations:** 1 National Research Council, Institute for Agriculture and Forestry System in the Mediterranean (CNR-ISAFoM), Rende, CS Italy; 2 National Research Council, Institute for Biomedical Research and Innovation (CNR-IRIB), Mangone, CS Italy; 3 National Research Council, Institute for Bioimaging and Biological Complex System (CNR-IBSBC), Catanzaro, Italy; 4 National Research Council, Institute of Translational Pharmacology (CNR-IFT), L’Aquila, Italy; 5 Emergency Medicine Department, Sant’Anna University Hospital, Cona, Ferrara, Italy; 6 Department of Medical and Surgical Sciences, University Magna Graecia of Catanzaro, Catanzaro, Italy; 7 Department of Science of Health, University Magna Graecia of Catanzaro, Catanzaro, Italy; 8 Department of Clinical and Experimental Medicine, University Magna Graecia of Catanzaro, Catanzaro, Italy; 9 Department of Pharmacy, Health and Nutritional Sciences, University of Calabria, Rende, CS Italy

**Keywords:** Plant extracts, Phytochemicals, Mitochondria, Mitochondrial DNA copy number, Mitochondrial function

## Abstract

Mitochondrial DNA copy number (mtDNA-CN) is a critical marker of mitochondrial health and plays a key role in cellular bioenergetics. Alterations in mtDNA-CN have been associated with aging, metabolic disorders and neurodegenerative diseases. Recent studies have revealed that various plant-derived extracts, as well as the secondary metabolites they produce, known as phytochemicals, can modulate mtDNA-CN through mechanisms including the regulation of mitochondrial biogenesis, oxidative stress, and mtDNA repair. This review examines plant-derived extracts and phytochemical compounds from a wide range of plant species- including *Ginkgo biloba*, *Crocus sativus*, Curcumin and many others- able to modulate mtDNA dynamics, scavenging oxygen free radicals and improving antioxidant defense systems.

## Introduction

### Mitochondrial genome: functions, integrity and maintenance

Mitochondria, highly dynamic double-membrane organelles present in almost all eukaryotic cells, are responsible for energy production via oxidative phosphorylation, preservation of Ca^2+^ ion homeostasis and modulation of programmed cell death. As known, mitochondrial ATP production may help the correct maintenance of cellular processes, and the synthesis of metabolites required for the generation of new macromolecules (Frezza et al. 2017). Numerous cellular functions, including cell development, communication, and death, are also mediated by mitochondria (Disha et al. 2024). Moreover, other activities sustained by these organelles and their functions, such as redox balance and heme biosynthesis, are closely linked to the integrity of mitochondrial DNA (mtDNA) (Vakifahmetoglu-Norberg et al. 2017). The presence of free mtDNA in the cytosol or in mitochondrial-derived vesicles (MDV) actives pathways including cyclic GMP-AMP synthase (cGAS) -stimulator of interferon genes (STING)—TANK binding kinase (TBK) and Toll-like receptor 9 (TLR9) (Peng et al. 2025). Moreover, mtDNA or MDV are transferred, secreted in the blood and, through some specific receptor, they enter in a cell (Phinney et al. 2015; Borcherding et al. 2022).

The preservation of the mitochondrial genome is crucial, because it lacks effective DNA repair systems and protective proteins such as histones. However, like all bacteria, mtDNA is condensed and packaged in a site of the cytosol named nucleoide (Shokolenko et al. 2015). In addition, mtDNA is localized near the oxidative phosphorylation (OXPHOS) machinery and DNA-damaging Reactive Oxygen Species (ROS) byproducts, thus it is more vulnerable to damage and accumulation of mutagenic events (Saravanan et al. 2022). Mitochondria are considered the "powerhouses" of eukaryotic cells and when they are unable to produce enough ATP, cells and tissues, that need a lot of energy, may malfunction (Ramaccini et al. 2021). Some cell types could "absorb" mitochondria or mtDNA from other cells. When the donated mitochondria are healthy, they can restore metabolic homeostasis in the recipient cells. This phenomenon contributes to intercellular mitochondrial transfer, a mechanism observed in tissue repair, immune modulation, and recovery from mitochondrial dysfunction. (Spees et al. 2006; Islam et al. 2012; Berridge et al. 2016; Torralba et al. 2016;).

The production of intermediates crucial for cell development would be inhibited if the oxidative phosphorylation machinery had flaws. Additionally, there would be an increase in ROS generation, dangerous for both mitochondrial and nuclear DNA, which would results in mitochondrial failure (Branco et al. 2023)**.** This occurs during the progression of cellular aging, which is a well-described biological process. Therefore, the preservation of mitochondrial functions in stem cells represents a powerful strategy to prevent aging and age-related pathologies. It has also been shown that mitochondria can be captured by some cell types to safeguard aerobic respiration and ensure cell division. Together, these findings have suggested that focusing on mitochondrial rescue could have an impact on disease progression (Borcherding et al. 2022).

### The role of mtDNA in the landscape of aging and diseases

It has been discovered that many diseases, including several neurodegenerative disorders, may exhibit a delay in mitochondrial dynamics (Chen et al. 2023a), as in the case of Parkinson's disease (PD), where aberrant fusion events happen (Pozo Devoto et al. 2017). These observations suggested that the maintenance of mitochondrial dynamics might be a strategy for controlling diseases linked to mitochondrial malfunction. The genetic material found in mitochondria, known as mtDNA, is maternally inherited and exists in multiple copies within each mitochondrion, endowed with the capability of self-replicating. The acquisition of new mutated genomes has been defined “heteroplasmy”, a condition in which a normal version of the maternal mtDNA genome and a version of the genome containing a mutation, simultaneously exist, as a result of both cells proliferation and differentiation (Nissanka et al. 2020; Citrigno et al. 2024). The coexistence of mutant and wild type mtDNA increases during aging and in age-related pathologies, and this accumulation leads to a worsening of clinical performance. On the other hand, many common pathologies converge towards the mitochondria, causing secondary mitochondrial dysfunctions, such as heart failure, neurodegeneration, metabolic syndrome, etc. (Wang et al. 2021; Eldeeb et al. 2022). The detection and management of abnormal quality, dysfunctional proteostasis, inhibited ATP production, calcium homeostasis, and metabolic reprogramming all occur simultaneously and interact within pathological conditions, affecting the mitochondria's ability to function as a biochemical hub. As a result, it is not surprising that mitochondrial dysfunction has been frequently linked to the physiopathology of many diseases and the aging process (Amorim et al. 2022; Picard et al. 2022).

### mtDNA-CN and its significance

mtDNA is organized as multiple mitochondrial DNA copy number (mtDNA-CN) per cells and its level is directly linked to genome stability, together with energy reserves, oxidative stress and changes in mitochondrial membrane potential (Castellani et al. 2020).

Mitochondrial content differs in each tissue based on proper energy demands and role. Several regulatory pathways, such as cellular and mitochondrial deoxynucleoside triphosphate (dNTP) pools, influence this heterogeneity. ATP synthase is a central sensor of the bioenergetics state and handles the copy number of mitochondria through the regulation of electrochemical potential and ROS. On the other hand, the expression of factors required for mtDNA stability get involved in the maintenance of mtDNA-CN levels under a normal range (Fukuoh et al. 2014). The correct management of these mechanisms could guarantee an adequate segregation during cell and mitochondria division and a functional mitochondrial ribosome biogenesis (Bendich et al. 1987). Although the deep efforts in investigating the role of nuclear-encoded genes and transcription factors involved in the regulation of mtDNA content, the way by which this complex machinery works in different tissues/organs are still poorly understood (Parchwani et al. 2025).

It has been proposed that molecular mechanisms underlying changes in mtDNA-CN content might be triggered in response to ROS/oxidative stress, accumulation of mutations into the mitochondrial genome and inflammation (Malik et al. 2013). Indeed, all these processes have an impact on changes in the quantity of mitochondrial mtDNA-CN, leading to the production of a destructive and dysfunctional loop (De Gaetano et al. 2021).

Because ROS are able to affect mitochondrial performance, they modulate mtDNA-CN levels through the activation of mitochondrial biogenesis, in order to compensate for the oxidative damage experienced by mitochondria. This hypothesis has been demonstrated in a study where the treatment of lung fibroblast cells (MRC-5) with H_2_O_2_ endorsed an increase of mtDNA content (Lee et al. 2000). Alterations of mtDNA-CN might also represent a consequence to the accumulation of mutations in regulatory regions or genes responsible for mtDNA replication/transcription apparatus, such as DNA polymerase subunit gamma (*POLG)*, Transcription Factor A, Mitochondrial (*TFAM)*, *p53* or Displacement- loop (*D-loop)* (Lin et al. 2019). It has been proposed that also inflammation could endanger mtDNA integrity. Under stress conditions, mtDNA can be fragmented and released from mitochondria, and it could act as damage-associated molecular patterns (DAMPs). These molecules are able to trigger the immune system, activating inflammatory pathways such as STING and toll-like receptors (TLRs). This process promotes the up-regulation of Interferon gamma (IFN-γ)-driven nuclear factor-kappa B (NF-kB) protein and increased ROS levels, responsible for mtDNA-CN alterations (Li et al. 2017).

It has been proposed that mtDNA content is also highly dependent by methylation/demethylation processes, the dynamic events which regulate the activity of mtDNA replication factors, such as TFAM and the D-loop region (Castegna et al. 2015).

Moreover, methylation of POLG, the nuclear-encoded mtDNA polymerase, can regulate the levels of mtDNA in several tissues, as well as mtDNA replication during neural differentiation and proliferation of cancer cells (Kelly et al. 2012; Lee et al. 2015).

Concurrently, the peroxisome proliferator-activated receptor gamma co-activator (PGC-1α) plays a crucial role in mitochondrial biogenesis, since a positive association between the hypermethylation of its promoter and mtDNA-CN levels has been reported (Bam et al. 2021).

Under typical physiological conditions, the cellular mtDNA-CN is essentially constant, and variations in this number can result in pathological alterations in tissues and organs. Indeed, because dysfunctional mitochondria produce several ROS, they can influence inflammation and oxidative stress. As a potential biomarker of mitochondrial dysfunction, mtDNA-CN has been linked to numerous diseases (Wang et al. 2025).

### Mitochondrial content-related diseases and the link with phytomedicine

Several physiological aspects seem to be related with mtDNA-CN activity, not only during adulthood but also in childhood or in fetal life, as the recognized capability to influence early developmental cell differentiation and reprogramming of induced pluripotent stem cells (Sun and St John 2016). Current evidence emphasizes the role of mtDNA-CN in the spectrum of aging processes, pinpointing how this parameter goes through several changes during adulthood, making it a critical contributor in predicting the development of aging-related diseases (Mengel-From et al. 2014; Castellani et al. 2020; Zhang et al. 2022; Lu et al. 2024). A decrease in mtDNA-CN is often observed not only in aging but also in neurodegenerative diseases, such as Alzheimer's disease (AD) and PD, where mitochondrial dysfunction contributes to the disease pathology (Lin and Beal 2006). In contrast, an increase in mtDNA-CN is typically a compensatory response to mitochondrial damage or stress, aiming at maintaining cellular energy production (Cerantonio et al. 2024).

An alteration in intracellular mtDNA-CN has been also associated with cancer, diabetes, and cardiovascular diseases (Sundquist et al. 2022). Although the exact mechanism by which fluctuations in mtDNA content could contribute to the pathogenesis of several diseases has not yet been clarified, some evidence suggests that changes in mtDNA content could play a crucial role in the development of such disorders, for which establishing a timely diagnosis and predicting disease progression is still demanding. Thus, the regulation of mtDNA-CN has emerged as a key therapeutic target for improving mitochondrial health and mitigating mitochondrial-related diseases (Wen et al. 2025).

Currently, there is renewed attention to herbal medicine and phytomedicine. In fact, nature-based therapy attracted modern society and pharmaceutical companies: the World Health Organization (WHO) estimated that about 4 billion people use herbal remedies for primary healthcare. This trend induced the release of strict regulations concerning efficacy, safety, and control of plant-based remedies (Ekor et al. 2014). Plants, in fact, have always represented a rich source of bioactive compounds, whose applications date back to very ancient times. A very recent literature proposed an interesting discussion concerning the role of natural products in targeting a wide range of mitochondrial dysfunctions, mainly associated with cancer, neurodegenerative and metabolic disorders. Thanks to their pleiotropic properties, broad activity, easy availability and the extraordinary potential to act as multitarget remedies, natural products, consisting in plant-derived extracts and phytochemicals, deserve a special attention in the modulation of mitochondrial dysfunctions (Chen et al. 2023b; Zhang et al. 2023; Anchimowicz et al. 2025). Additionally, as previously discussed, the key and even increasingly central role of mtDNA-CN lead to wondering how plant-derived extracts and phytochemicals are directly or indirectly able to modulate them. In fact, although mutations of mtDNA copies are strictly connected with the onset of diseases, current evidence has demonstrated that also the absolute value of mtDNA-CN matters (Filograna et al. 2021).

In this context, this work provides a comprehensive overview of the phytochemicals and plant extracts derived from various plant species influencing mtDNA-CN. We explored their therapeutic potential in conditions characterized by mitochondrial dysfunction. In the following sections, the beneficial effects of phytochemicals targeting mitochondria will be examined, with an emphasis on the most recent findings. Lastly, a few perspectives on this topic will be addressed and the possible role of phytochemicals and plant extracts in cancer onset, metabolic and neurodegenerative diseases will be discussed.

## Methods

### Study selection

For this review, a comprehensive search of scientific literature from 2000 to 2024/2025 has been done. In this time frame, due to the increasing number of articles published by scientific journals, we focused on research investigating the effects of plant extracts and phytochemicals which directly impact on the amount of mtDNA-CN, in terms of increase or decrease. Studies were identified through searches in major bibliographic databases, including PubMed, Scopus, Google Scholar, and Web of Science, using keywords such as, “plant extracts”, "phytochemicals", “phytochemical compounds”, “plant secondary metabolites", mtDNA copy number", "mitochondrial function".

Boolean operators (AND, OR) have been employed to improve our bibliographic research, optimizing the selection of the most relevant articles on this topic. Inclusion criteria required that studies had to be written in English, published in peer-reviewed journals and focused on the effect of plant extracts or phytochemicals on mtDNA or mitochondrial function, providing quantitative data on mtDNA-CN or other measures of mitochondrial integrity. Moreover, studies had to display mechanistic insights on how the identified biomarkers affected liver function.

Selected studies had also to employ cellular and animal models, in addition to those conducted in humans, to better examine the regulation of biomarkers associated with liver damage and to provide quantitative data regarding their expression and correlation with histological and functional parameters of the liver. The inclusion of these studies allowed us to accurately identify not only the pathogenic mechanisms, but also the potential diagnostic and prognostic indicators of liver diseases.

Studies have been chosen only when they clearly reported the type of plant extracts or phytochemicals investigated, and the mechanisms involved in the interaction between plant extracts or phytochemicals and mtDNA-CN. Moreover, we favored the selection of papers highlighting changes in mtDNA-CN or mitochondrial function after treatment with plant extracts or phytochemicals.

Additionally, particular emphasis has been given to studies relying on advanced molecular techniques, such as quantitative Real-Time PCR (qPCR) and next-generation sequencing (NGS), to ensure data accuracy and reproducibility.

Review articles, conference abstracts and studies lacking molecular evidence of biomarkers associated with hepatic steatosis have been excluded.

Only papers including a study design and describing the type of biomarkers considered, the systems used and the key results obtained have been included in our review.

All the figures used to depict the main pathways described in this review have been generated using Biorender Software.

## Plant extracts and their bioactive compounds

Phytochemicals and plant extracts have been studied for their ability to influence mitochondrial function and regulate mtDNA-CN (Table 1). These plant-based compounds typically exert their effects through antioxidant activity, the regulation of mitochondrial biogenesis, and the activation of pathways involved in mitochondrial health.

**Table 1:** Plant extracts and phytochemicals modulating mtDNA-CN

Plant extracts	Phytochemicals	Model	Conducted study	Effective dose	Effect on mtDNA-CN	Reference
	curcumin from *Curcuma longa*	*Caenorhabditis elegans*	Animal model	25 µM	Increase	(Xu et al. 2023)
	Gingkolic Acids from *Ginko biloba*	HeLa cells	in vitro	50 µM	Decrease	(Wang et al. 2019)
	Ginsenosides	Muscle cells and neurons	in vitro	-	Increase	(Huang et al. 2021a)
*Panax ginseng*		62 men affected by metabolic syndrome 63 post- menopausal women Obese Type 2 mice models	Randomized, double-blind, placebo controlled study Double-blind placebo controlled clinical trial Animal model	3.0 g/day for 4 weeks 2 g of KGR tablet/day. Tablet prepared by dehydrating 3 g of KGR extract 100 mg/kg for 12 weeks	Increase Increase Increase	(Jung et al. 2016) (Chung et al. 2021) (Park et al. 2018)
*Morus alba* (mulberry and mulberry wine extracts)	Cyanidin-3- glucoside	C_3_H_10_T_1/2_ mesenchymal stem cells C_3_H_10_T_1/2_ mesenchymal stem cells	in vitro in vitro	10 µg/mL for 6 days 10 µM	Increase Increase	(You et al. 2015) (You et al.^ 2015^)
*Fuzi (Aconitum carmichaeli roots)*	Benzoylaconine	HepG2 cells HepG2 cells	in vitro in vitro	10 mg/mL 25, 50, 75 µM	Decrease Increase	(Deng et al. 2019a) (Deng et al. 2019a)
*Crocus sativus*		40 8-week aged male Wistar rats	Animal model + endurance training	40 mg/kg	Increase	(Akbari- Fakhrabadi et al. 2019)
	Oligonol from lychee fruit and green tea	4- week- old male diabetic C57BLKS/J (*db/db)* mice	Animal model	20 mg for 10 weeks	Increase	(Liu et al. 2016)
	Silybin and Silychristin from *Silybum marianum*	A549 cells	in vitro	50, 100 µM	Decreased mtDNA damage	(Bijak et al. 2017)
	Cyclovirobuxine D from *Buxus* *microphylla*	Adult C57BL mice (18–22 g)	Animal model	1 mg/kg/d for 4 days	Preservation	(Guo et al. 2015)
	Procyanidin B2 from grape seed	Podocytes Glucosamine- stimulated rat mesangial cells	in vitro in vitro	2.5, 5, 10 µg/mL 10 mg/mL	Increase Increase	(Cai et al. 2016) (Bao et al. 2015)
*Zingiber officinale*	6-gingerol	Mice HepG2 cells CTLL-2 cells HepG2 cells CTLL-2 cells	Animal model in vitro in vitro in vitro in vitro	2 g/kg 2.5, 5 mg/mL 5, 10 mg/mL 25, 50, 100, 200 µM 50, 100, 150 µM	Increase Increase Increase Increase Increase	(Deng et al. 2019a; Deng et al. 2022)
	Lycopene	Intestinal porcine enterocytes (IPEC-J2 cells) SH-SY5Y cells	in vitro in vitro	Lycopene 10 µM Lycopene 10 µM + Deoxynivalenol 1 µg/mL Lycopene 2 µM + 500 µM MPP	Increase Preserve	(Wang et al. 2024) (Yi et al. 2013)
	Berberine	K1735-M2 mouse melanoma cells	in vitro	25 µM	Decrease	(Pereira et al. 2007)
	(-)-Epigallocatechin-3-gallate	Senescence accelerated mice prone 8 (SAMP8)	Animal model	3.2 g/kg chow diet for 12 weeks	Restore levels	(Liu et al. 2015)
	Flavan-3—ol from cocoa	Mice	Animal model	50 mg/kg for 2 weeks	Increase	(Watanabe et al. 2014)
	Halimane, Labdane from *Plectranthus ornatus*	MCF7, Fadu cells	in vitro	IC50 Halimane: 15.12 µg/mL IC50 Labdane: 32.66 µg/mL	Decrease	(Sitarek et al. 2022)
*Tithonia diversifolia*		Rats	Animal model	100 mg/kg for 28 days	Increase	(Istikharah et al. 2022)
Cannabis		19–25 years adults	Double-blind, placebo- controlled, parallel groups, Randomized clinical trial	Cigarettes, 750 mg of Cannabis (12.5% Δ9-THC) 1–4 days/week	Decrease	(Powlowski et al. 2025)
	Resveratrol	C57BL/6N mice Porcine oocytes	in vitro in vitro	0.1 mM Resveratrol 20 µM + MG132 10 µM	No differences Increased in blastomers of blastocysts	(Teramoto et al. 2024) (Sato et al. 2014)
*Albizia julibrissin*		3T3-L1 cells	in vitro	1, 10, 50 µg/mL	Increase	(Kim et al. 2023)
*Moringa oleifera*		24 Sprague–Dawley rats	Animal model	200,400 mg/kg + 4 mg/kg Doxorubucin	Increase	(Patintingan et al. 2023)
*Ephedra sinica*		Mouse inguinal pre- adipocytes (miPA); human adipose- derived stem cells	in vitro	10 µg/mL in miPA differentiated cells	Increase	(Park et al. 2022)
*Punica granatum*		Ca9—22, HSC-3, OC-2 cells	in vitro	0, 50, 100 µg/mL	Decrease	(Peng et al. 2021)
	Oregonin	Embryonic, MEF and NIH/3T3 cells	in vitro	50, 100 µM	Increase	(Krasilnikova et al. 2018)
	Apigenin	60 male C57BL/6 mice	Animal model	25, 50, 100 mg/kg per day	Increase	(Wang et al. 2020)

## Curcumin from* Curcuma longa*

Curcumin is a naturally occurring pigment approved as a food additive, traditionally isolated from *Curcuma longa* rhizomes and well-known for the wide range of activities it exerts. Curcumin is considered a strong antioxidant, able to protect mitochondrial damages by limiting oxidative stress and lipid peroxidation and by exerting anti-inflammatory properties, preserving from cardiovascular risk. In a study conducted by Xu and coworkers, 10, 25, 50 and 100 µM of curcumin, respectively, were administered in *Caenorhabditis elegans,* an invertebrate animal model*.* Results evidenced that treated groups showed a significantly longer lifespan if compared to the control group (not treated) (Xu et al. 2023).

Curcumin decreased ROS levels in models previously treated with Paraquat or subjected to heat exposure: data showed that at the concentration of 25 µM, curcumin increased mtDNA-CN in nematode, enhancing its longevity and preserving mitochondrial integrity by downregulating Mitogen-activated protein kinase (MAPK) Signaling Pathway, as emerged by RNA-seq analyses. (Xu et al. 2023). In addition, Zhang and coauthors investigated the effects of dietary curcumin supplementation on the modulation of mitochondrial redox system, mtDNA integrity, and antioxidant-related gene expression in the liver of broiler chickens. Curcumin mitigated mitochondrial dysfunction in heat stressed broilers by suppressing ROS burst, maintaining both the thiol pool and the mtDNA content, and enhancing mitochondrial antioxidant gene expression (Zhang et al. 2018).

## Ginkgolic acids from *Ginkgo biloba*

Extracts from *Ginkgo biloba,* extensively studied for their neuroprotective effects, are gaining attention and appreciation as mitochondrial modulators. The plant active compounds, ginkgolides and flavonoids, are considered potent antioxidant molecules, able to reduce oxidative stress. *Ginko biloba* is traditionally used in Chinese medicine for the treatment of memory and cognitive damages. Ginkgolic Acids (GA), commonly extracted from *Ginko biloba* leaves, nuts and testa, not only exert specific antitumor and antibacterial properties, but are also able to modulate mitochondrial functions. Hypothesizing on this latter effect, Wang et al. (2019) investigated the activity of 25 and 50 µM of GA treatment in HeLa cell lines. Mitochondrion-related biological processes appeared significantly changed in the GA treated group if compared to Dimethyl Sulfoxide (DMSO) control group at gene ontology analyses. Moreover, immunofluorescent staining revealed that GA treatments affected mitochondria morphology by inducing fragmentation and reducing mitochondrial mass. Then, to establish if the reduced mitochondrial mass affected mitochondrial biogenesis, mRNA levels of several different proteins were analyzed. GA treatment significantly decreased PGC-1α levels, while treatment with ZLN005, a PGC-1α antagonist, restored mitochondrial protein loss. These findings indicated that GA treatment reduced mitochondrial mass by arresting mitochondrial biogenesis; suggested mechanisms included modulation of PGC-1α protein levels and mitophagy.

As a result, this study demonstrated that GA treatment (50 µM, 24 h) caused mitochondrial fragmentation and reduced mtDNA-CN, compared to DMSO group. Consistently, it is important to underline that GA treatment significantly affected cell viability in the selected cell model, impaired mitochondrial protein levels, and damaged mitochondrial adenosine 5’-triphosphate production and oxygen consumption (Wang et al. 2019).

### Panax ginseng

*Panax ginseng* is a traditional adaptogen plant species with demonstrated activity in mitochondrial function modulation. Active compounds such as ginsenosides have been found to increase mtDNA-CN in muscle cells and neurons (Huang et al. 2021a).

Starting from evidence that mitochondrial dysfunctions are strictly related to an increased risk of metabolic syndrome, Jung et al. (2016) investigated the effects of Red Ginseng (RG) in 62 men affected by this disorder. Individuals recruited for a randomized, double-blind, placebo-controlled study were treated with RG (3.0 g/day) or placebo group for 4 weeks and, different parameters, including inflammatory markers, hormones, leukocyte, mtDNA-CN were evaluated. As a result, men treated with RG showed a significant amelioration in mitochondrial function (95% CI −44.9 to −1.3) if compared to placebo group, as well as a reduction in blood pressure, serum cortisol and an increase of total testosterone levels (Jung et al. 2016).

Chung et al. (2021) evaluated the effects of Korean Red Ginseng (KRG) on aging and antioxidant capacity in post-menopausal women. To this aim, 63 participants were involved in a double-blinded placebo-controlled clinical trial: KRG in tablet (2 g/day) and placebo were randomly administered: both mtDNA-CN and total antioxidant status (TAS) were evaluated. Administered KRG tablets were prepared by dehydrating 3 g of KRG extract (standardized in ginsenoside, 8.03 mg/g) per 2 g of tablets, while placebo tablets contained cornstarch, cellulose and the same flavors of KRG tablets, only. TAS was investigated, DNA was extracted and mtDNA-CN was measured by real-time PCR. Results revealed that KRG significantly increased mtDNA-CN, total antioxidant activity, and symptoms correlated to postmenopausal syndrome. These findings suggested that KRG could offer protection against aging, increase cellular metabolism and reduce symptoms related to fatigue in post-menopausal women (in which estrogen levels, usually offering protection against mitochondrial apoptosis and oxidative stress, are significantly lower if compared to pre-menopausal women) (Chung et al. 2021). According to previous studies, the possible mechanisms of action discussed by authors included that KRG improve PGC-1α-Nuclear Respiratory factor 1 (NRF1)-TFAM pathway (Taherzadeh- Fard et al. 2011; Gureev et al. 2019), antioxidant indicators such as Superoxide Dismutase (SOD), Glutathione Peroxidase (GPx), catalase, decrease oxidative stress markers (Kim et al. 2011; Ramesh et al. 2012; Yu et al. 2014) and decrease fatigue symptoms (Bach et al. 2016). Park and coauthors performed another study about KRG in animal models with type 2 diabetes mellitus (T2DB) suggesting that its antidiabetic effect could be mediated by protection of mitochondrial dysfunction and intracellular inflammation. Obese type 2 diabetic mice models were treated with KRG (100 mg/kg) for 12 weeks, then metabolic parameters and skeletal muscle tissue were analyzed by means of High-performance Liquid Chromatography (HPLC) and quantitative real-time PCR (qPCR) analyses, respectively. Data showed that mice treated with KRG presented a significant reduction of cholesterol, insulin, low-density lipoprotein and an increase in mtDNA-CN, suggesting that the extract protective effects on metabolism could be due to mechanisms of mitochondrial function protection (Park et al. (2018).

## Mulberry, mulberry wine extracts and Cyanidin-3-glucoside

Mulberry extracts, produced by *Morus alba* L. fruits, are rich in water-soluble anthocyanins, active principles known for their antioxidant properties and exhibiting a large spectrum of biological activities, including antibacterial, anti-inflammatory and antioxidant properties. It is already known that, in glioma cells, mulberry fruit extract influences mitochondrial membrane potential (MMP), probably through the modulation of ROS-dependent mitochondrial pathway (Jeong et al. 2010). A study conducted by You and coworkers, demonstrated that mulberry and mulberry wine extracts (10 µg/mL for 6 days) modulate the activity of brown adipose tissue (BAT) and mitochondrial functions in adipogenesis. BAT, due to its intrinsic characteristics of being a thermogenic organ, is characterized by a high number of mitochondria. Here, the treatment with both mulberry and mulberry wine extracts, significantly increased the expression of two key-players in mitochondrial biogenesis, TFAM and NRF1, as wells as the expression of Uncoupling Protein 1 (UCP-1), PGC1α, PR domain containing 16 (PRDM16) and the OXPHOS proteins present on the mitochondrial membrane during adipogenesis. Moreover, extracts increased the mitochondrial number in C_3_H_10_T_1/2_ cells. Cyanidin-3-glucoside (10 µM) significantly increased mtDNA-CN, ameliorated cellular oxygen consumption and fatty acids oxidation related genes in brown adipogenesis (You et al. 2015).

### Aconitum carmichaeli

*Fuzi* (*Aconiti Lateralis Radix Preparata*), produced by *Aconitum carmichaeli* Debx roots, is a traditional Chinese medicine, linked to the Yin-Yang theory, and known for its anti-inflammatory, antitumor, analgesic and immunostimulant activities. *Fuzi* and some metabolites such as Aconitine, Benzoylaconine and Aconine, are alkaloids already known for their ability to increase mitochondrial mass. *Fuzi* extract (10 mg/mL) and the three above-mentioned secondary metabolites were screened in HepG2 cell model: data showed that only Benzoylaconine increased mitochondrial mass in a dose-dependent manner. Moreover, Benzoylaconine at 25, 50 and 75 µM increased mtDNA-CN of 34, 27 and 30%, respectively, and ameliorated ATP production of 22% at the highest tested concentration (75 µM) (Deng et al. 2019a).

### Crocus sativus

*Crocus sativus,* together with Saffron, the flower dried stigma, mainly known for their culinary properties, showed remarkable biological activities.

In a study conducted by Akbari-Fakhrabadi et al. (2019), a saffron hydroalcoholic extract obtained through ultrasonication was administered in Wistar rats to observe endurance training on mitochondrial biogenesis. Forty male rats were divided into four groups including saffron, exercise + saffron, exercise + placebo and placebo only; metabolic indicators were evaluated after 8 weeks. Rats belonging to exercise + saffron 40 mg/kg group showed a statistically significant increase in both mtDNA-CN and *NRF-1* gene expression if compared to the placebo group. Moreover, parameters such as Aspartate Aminotransferase (AST), Creatine Phosphokinase (CPK), Interleukin-6 (IL-6) and malondialdehyde decreased, while antioxidant indicators like glutathione and GPx significantly increased in the saffron + exercise group, showing that the extract improved antioxidant activity, anti-inflammatory pathways and ameliorated mitochondrial biogenesis (Akbari-Fakhrabadi et al. 2019).

## Oligonol from lychee fruit and green tea

Oligonol is a phenolic compound traditionally extracted from lychee fruit and green tea, which dietary supplementation is associated with lowered glucose and insulin levels and better oral glucose tolerance. Liu and coworkers investigated the effects of oligonol on kidney damage in diabetic db/db mice, demonstrating that this secondary metabolite (20 mg for 10 weeks) not only reduced inflammatory cytokines (IL-6) and pathways including NF-kB and renal oxidative stress, but also significantly increased mtDNA-CN and biogenesis by up-regulating NRF1 and TFAM. All these findings suggested that Oligonol could be considered effective in protecting kidney against damages induced by diabetes (Liu et al. 2016).

## Silybin and Silychristin, flavonolignans from *Silybum marianum*

Milk thistle (*Silybum marianum*), also called “wild artichoke” is a plant with a very long traditional use as a natural remedy for the treatment of liver and gallbladder diseases. Bijak and coauthors evaluated the cytotoxic and genotoxic effects of some *Silybum marianum* secondary metabolites, such as flavonolignans, in different cell models. As a result, none of tested active principles, Silybin, Silychristin, Silydianin exerted cytotoxicity and genotoxicity at concentrations up to 100 µM on blood platelets, lung cancer cell line (A549) and peripheral blood mononuclear cells (PBMCs). Moreover, both Silybin and Silychristin showed efficacy in reducing spontaneous mtDNA damages in A549 cell model (detected through mtDNA copies calculation), and mtDNA lesion in nicotinamide adenine dinucleotide hydrogen (NADH) dehydrogenase 1 (*ND1*) and nicotinamide adenine dinucleotide hydrogen (NADH) dehydrogenase 5 (*ND5*) genes. These findings highlighted the potential cellular mitochondrial protective role of flavonolignans (Bijak et al. 2017).

## Cyclovirobuxine D (CVB-D) from *Buxus microphylla*

Cyclovirobuxine D (CVB-D), extracted from *Buxus microphylla*, is a triterpenoid alkaloid traditionally known in Chinese medicine for its cardiovascular protective effects on arrhythmias, heart failure and myocardial ischemia. Guo and coworkers employed C57BL adult mice to investigate the cardioprotective effects of CVB-D in combination with Doxorubucin, an anthracycline antibiotic used in the treatment of solid and hematological tumors, which beneficial activity results significantly compromised for its cardiotoxic side effects. Results showed that CVB-D 1 mg/kg/day for 4 days pretreatment reduced Doxorubicin-induced cardiomyopathy by preserving mitochondrial biogenesis through PGC1-α, NRF-1 and mtDNA-CN and by decreasing oxidative damage (Guo et al. 2015). Furthermore, Jiang et al. (2020) investigated the effects of CVB-D in a rat model of diabetic cardiomyopathy (DCM) induced by a high-fat, high-sucrose diet combined with streptozotocin. This study demonstrated that CVB-D holds potential as a therapeutic agent for DCM by activating the Nuclear Factor Erythroid 2- Related Factor 2 (Nrf2) signaling pathway and mitigating oxidative stress (Jiang et al. 2020). These findings are further supported by Gao and coworkers: they reported that CVB-D significantly ameliorates DCM by reversing cardiac dysfunction and associated pathological changes. The underlying molecular mechanism involves CVB-D's ability to inhibit cardiomyocyte pyroptosis through the NOD-, LRR- and pyrin domain-containing protein 3 (NLRP3) inflammasome pathway, thereby mitigating inflammation and cell death (Gao et al. 2022).

## Grape seed procyanidin B2

Procyanidin B2, a natural edible pigment available in food, is commonly isolated from grapes. Cai and coworkers studied the in vitro effects of grape seed procyanidin B2 (GSPB2), a molecule already known for its ability to protect against diabetic nephropathy by exerting antioxidant activity. Procyanidin B2, at the concentration of 10 µg/ml, suppressed podocyte apoptosis and mitochondrial dysfunction induced by high glucose level, ROS production and oxidative stress through the modulation of nephrine, podocalyxin, NRF-1, TFAM, mtDNA-CN and AMPK-Sirtuin 1 (SIRT1) -PGC-1α axis (Cai et al. 2016). Bao and coauthors investigated the effects of grape seed procyanidin B2 in the treatment of diabetic nephropathy through a high dose glucosamine-stimulated rat mesangial cells model. As a result, they obtained that treatment with grape seed procyanidin B2 (10 mg/mL) in mesangial cells, previously stimulated with glucosamine 15 mM, significantly increased cell viability, inhibited apoptosis and decreased oxidative stress through stimulation of GPx and SOD enzymes activity. Moreover, GSPB2 induced both NRF-1 and TFAM expression, and increased mtDNA-CN through a protective effect probably exerted by AMPK-SIRT1-PGC1α axis activation (Bao et al. 2015).

## *Zingiber officinale* extract and its main active compound 6-Gingerol

*Zingiber officinale* Roscoe is a perennial, herbaceous plant traditionally used all over the world as food spice. Ginger extract (GE) and its main active phytochemicals, 6-Gingerol and 6-Shogaol, were investigated for their effects on mitochondrial biogenesis, starting from the experimental evidence that this plant regulate thermogenesis and energy expenditure, activities strictly correlated with mitochondrial function. Deng and coworkers demonstrated that the administration of GE, 2 g/kg, in mice, increased oxygen consumption and mtDNA-CN both in muscle and liver. Moreover, an increased expression of OXPHOS proteins and an upregulation of AMPK/PGC1α signaling pathway was observed in muscle, liver, and brown adipose tissue after treatment with extract. Both GE (concentrations of 2.5 and 5 mg/mL) and 6-Gingerol, one the most abundant compound in *Zingiber officinale* extract, detected through HPLC analyses at doses of 25, 50, 100 and 200 µM, respectively, improved mitochondrial mass, mtDNA-CN and ATP production in HepG2 cell line. Moreover, this work demonstrated that 6-Gingerol, by activating AMPK-PGC-1α pathway, modulated mitochondrial biogenesis and overall mitochondrial functions. These findings suggested that GE extract was effective in the promotion of mitochondrial biogenesis and function via modulation of AMPK-PGC-1α pathway and that 6-Gingerol, alone, was responsible for the same biological activities, leading to the conclusion that the pure single molecule is the main active compound of GE extract. It is important to underline that 6-Gingerol, at the highest tested dose, 200 µM, affected HepG2 cell viability up to 11% (Deng et al., 2019). In 2022, the same research group investigated GE and 6-Gingerol anticancer properties by protecting mitochondrial biogenesis in tumor infiltrating CD8 + T cells. Data showed that GE (5 and 10 mg/mL) and 6-Gingerol (50, 100, 150 µM), both increased mtDNA-CN in CTLL-2 cells (Deng et al. 2022). It has also been proposed that 6-Gingerol can activate downstream AMPK signaling pathways via Adiponectin Receptor 1 (AdipoR1), thereby enhancing mitochondrial function and improving lipid metabolism disorders in skeletal muscle (Peng et al. 2024).

## Lycopene

Lycopene, a pigment widely distributed in tomatoes, watermelons and carrots, is known for the high antioxidant activity expressed as free radical scavenger power and ability to quench singlet oxygen species. Previous studies showed that Lycopene was able to protect broilers from hepatic mitochondrial damages and dysfunctions caused by aflatoxin B1. Wang and colleagues verified the effects of lycopene 10 µM in intestinal porcine enterocytes (IPEC-J2) cells after exposure to Deoxynivalenol (DON), a mycotoxin largely diffused in food and feed. Results showed that Lycopene attenuated cell damages and apoptotic response, and improved mitochondrial function by increasing MMP, mtDNA-CN and ATP reserves. Moreover, lycopene treatment of IPEC-J2 cells after DON exposure significantly decreased ROS content and regulated oxidative stress levels by increasing SOD, catalase (CAT), and lactate dehydrogenase (LDH). The suggested mechanism of action involved the activation of the OXPHOS signaling pathway (Wang et al. 2024).

Yi and coworkers investigated the Lycopene biological activity in dopaminergic neuronal death induced by toxins. SH-SY5Y cells were treated with lycopene (2 µM, 2 h) and stimulated with PD-1-methyl-4-phenylpyridinium iodide (MPP(+)) 500 µM (24 h): data showed that lycopene not only decreased apoptotic rate, but also increased cell viability and attenuated MMP(+) induced toxicity and was effective in suppressing both ROS and lipid peroxidation. Lycopene decreased ROS production, attenuated morphological changes, increased membrane potential, and reversed both the reduced ATP levels and the mtDNA-CN promoted by MMP(+) exposition in mitochondria. In conclusion, these evident protective effects candidate lycopene as a promising therapeutic strategy for PD, which pathogenesis seems to be strictly correlated to mitochondrial dysfunction (Yi et al. 2013). Furthermore, due to the limited bioavailability and inefficient endocytosis of Lycopene across the Blood–Brain Barrier (BBB), its concentration within the neurons remains low, thereby restricting its neuroprotective effects. To address this challenge, Xia and coworkers designed and synthesized rationally sequenced, targeted lycopene nanodots, which facilitate neural enrichment and mitochondrial regulation of lycopene via BBB transcytosis and neuronal mitochondria-targeting capabilities. These nanodots could restore Parkinsonian motor symptoms in a mouse model of PD and could be considered a promising approach for the treatment and prevention of this disease (Xia et al. 2023).

## Berberine

Berberine, a naturally occurring alkaloid with a long traditionally history in Chinese and Native American medicine, is well known for its ability to arrest cell cycle and induce apoptosis in many different cancer cell lines. Pereira and colleagues investigated the effects of Berberine in K1735-M2 mouse melanoma cells. In order to assess viability, cells were treated with different Berberine concentrations (ranging from 5 to 100 µM) for 24, 48, 72 and 96 h. The molecule showed a time-dependent effect, with 50% inhibition of cell growth at 72- and 96-h exposure. Only one concentration, 25 µM, was selected and carried forward for further investigation: as a result, epifluorescence microscopy showed that Berberine selectively accumulated in mitochondria of melanoma cells after 6 h of exposure. Berberine also induced alterations of MMP and morphology, described as mitochondrial fragmentation after 6 h only. It also increased oxidative stress and ROS production on isolated mitochondria, and decreased ATP levels without affecting ATPase activity. mtDNA-CN content was evaluated through real-time PCR: the highest tested dose, 100 µM, did not modulate mtDNA-CN, while the lowest dose, 25 µM, decreased it. The unchanged modulation of CN at high doses may be explained by a compensatory mechanism that tries to overcome the decreased mitochondrial ATP production by increasing mtDNA-CN. Alternatively, the high oxidative stress produced by low concentrations of Berberine can lead to the destruction of mtDNA (Pereira et al. 2007).

All these findings are further supported by Sajeev and coworkers and suggested that besides the interesting chemotherapeutic properties of Berberine, its toxicological profile needs attention and evaluation before high-dose administration (Sajeev et al. 2024).

## (-)-Epigallocatechin-3-gallate

(-)-Epigallocathechin-3-gallate (ECGC) is the most abundant catechin in green tea, which dietary supplementation is proved to ameliorate insulin sensitivity and fatty liver disease. ECGC 3.2 g/kg chow diet for 12 weeks supplementation reduced blood glucose and insulin levels by replacing serine/threonine Protein Kinase B (Akt), Glucose Transporter Type 4 (GLUT4) and AMPK activation in muscle. It stimulated mitochondrial biogenesis and increased mtDNA-CN in skeletal muscle of senescence-accelerated mice prone 8 (Liu et al. 2015).

## Flavan-3—ol fraction from cocoa powder

Flavan-3—ol group represents a pull of polyphenolic compounds including flavan-3—ol monomers, (+)-catechin, (-)-epicatechin, oligomers and procyanidins type B with C4-C8 bond, largely spread in cocoa, wine and apples. Watanabe and colleagues investigated the effects of flavan-3—ol extracted from cocoa: 50 mg/kg of this fraction were administered in mice for 2 weeks. Treated mice showed a decreased respiratory exchange ratio and blood pressure. Carnitine, palmitoyltransferase 2 and UCP-1 significantly increased, as well the mtDNA-CN in gastrocnemius and soleus muscles and in BAT, suggesting that flavan-3—ol fraction from cocoa powder promoted lipolysis and mitochondrial biogenesis (Watanabe et al. 2014). Moreover, supplementation with flavan-3-ols significantly reduced blood pressure and enhanced endothelial function (Godos et al. 2014).

## *Plectranthus ornatus* derived phytochemicals

Halimane (11R*,13E)−11-acetoxyhalima-5,13-dien-15-oic acid (HAL) and the labdane diterpenes 1α,6β-diacetoxy-8α,13R*-epoxy-14-labden-11-one (PLEC) and forskolin-like 1:1 mixture of 1,6—di-*O*-acetylforskolin and 1,6—di-*O*-acetyl-9-deoxyforskolin (MRC) isolated from *Plectranthus ornatus* were tested in MCF-7 and FaDu, two cancer cell lines. Isolated phytochemicals (Halimane IC50 15.12 µg/ml and Labdane IC50 32.66 µg/ml) induced apoptosis in both cellular models, increased ROS production, decreased MMP, mtDNA-CN, and modulated several pro- and anti-apoptotic genes (Sitarek et al. 2022).

### Tithonia diversifolia

*Tithonia diversifolia* (Hemsley) A Gray, a plant species already known in scientific literature for its ability to control hyperglycaemia, was investigated by Istikharah and colleagues for the effects on insulin resistance and mtDNA-CN, starting from the assumption that changes in mtDNA-CN can protect from mitochondrial damages caused by insulin resistance-induced oxidative stress. Leaves were processed by ultrasound-assisted extraction, and the obtained extract was standardized in Tagitinin C, according to the Indonesian National Agency of Drug and Food Control requirements. Experiments were performed on rats previously divided into 6 groups: control, diabetic metformin (300 mg/kg) and 3 different extract concentrations (50, 100 and 150 mg/kg) groups. Blood samples (time 0 and after 28 days), soleus and gastrocnemius muscles were collected after 28 days for mtDNA-CN evaluation. Results showed that animals treated with *T. diversifolia* extract (100 mg/kg) had a significant increasing number of mtDNA-CN (up to 3 times) in soleus muscles. All these findings suggested this extract exerted an anti-diabetic activity by improving insulin-resistance and mtDNA-CN (Istikharah et al. 2022).

## Cannabis

In 2020, Cannabis was considered the third most common used psychoactive drugs worldwide, after alcohol and tobacco. Its toxic effects are mainly attributed to Δ9-tetrahydrocannabinol (Δ9-THC), rapidly adsorbed in lungs and promptly detected in blood. Several different studies correlated the interaction between mitochondria and Δ9-THC with altered memory and metabolism in mice. Powlowski and colleagues established a double-blind, placebo-controlled, parallel-group randomized clinical trial in which blood samples from adults (19—25 years) usually consuming Cannabis received randomly cigarettes containing 750 mg of Cannabis (12.5% Δ9-THC) and placebo (< 0.01% Δ9-THC) 1—4 days/week. As a result, this study highlighted that the consumption of active Cannabis was correlated with a reduction in mtDNA-CN in blood both 15 and 60 min after smoking. The presence of mtDNA-CN was inversely proportional to 11-hydroxy-Δ9-tetrahydrocannabinol (11-OH-THC) and 11-Nor-9-carboxy-Δ9-tetrahydrocannabinol (THC-COOH), the two primary metabolites of Δ9-THC, even if this effect was observed only in a small group of people (Powlowski et al. 2025).

## Resveratrol

Resveratrol, a polyphenol commonly found in grapes, peanuts, berries and cocoa, is well known as an antioxidant, anti-aging and anti-inflammatory agent. It was reported that Resveratrol promotes mitochondrial biosynthesis and degradation in embryos and oocytes by activating Silent mating type information regulation 2 homolog (SIRT1). Teramoto and coworkers investigated the mtDNA-CN and telomere length in blastocysts produced by mice. Water and water supplemented with Resveratrol 0.1 mM were administered to C57BL/6N male mice used for embryo production. Real-time PCR revealed no difference in mtDNA-CN in sperm. Anyway, Resveratrol consumption increased mtDNA-CN and telomere length in blastomer of blastocysts coming from old father but did not modulate these factors in embryos derived from young mice (Teramoto et al. 2024).

Sato and coworkers investigated how Resveratrol influenced SIRT1 expression and mitochondrial activity in porcine oocytes. A supplementation of 10 µM MG132, a proteasome inhibitor, in the maturation medium, increased mtDNA-CN by 14%, while the addition of Resveratrol 20 µM to this mixture increased mtDNA-CN by 39%. Moreover, data showed that SIRT1 expression and copy number in oocytes were strictly correlated (Sato et al. 2014). In their recent study, Gureev and coworkers investigated mitochondrial damage and the effects of Resveratrol on inflammation, cognitive function, and mitochondrial quality control in APP/PS1 mice. They demonstrated that Resveratrol had the potential to attenuate inflammation and improve mitochondrial quality control; however, it did not reduce amyloid-β (Aβ) levels (Gureev et al. 2025).

### Albizia julibrissin

*Albizia julibrissin*, the common mimosa or Persian silk tree, is traditionally used for insomnia, amnesia, sore throat and contusions. *A. julibrissin* leaf extract was investigated for its potential anti-obesity properties in 3T3-L1 cell model. Kim and colleagues showed that the extract was able to reduce adipogenesis process through the inhibition of genes involved in fat synthesis, peroxisome proliferator-activated receptors gamma (PPARγ), CCAAT/enhancer binding proteins (C/EBPs) and the decrease of lipid droplets and triglycerides production. Moreover, *A. julibrissin* leaf extract induced the expression of TFAM, stimulating mtDNA replication and, therefore, increased mtDNA-CN at 1, 10 and 50 µg/ml, respectively (Kim et al. 2023).

## *Moringa oleifera* leaf extract

A commercially available *Moringa oleifera* leaf extract was investigated by Patintingan and colleagues for its cardioprotective effects against Doxorubicin-induced damages. 24 Sprague–Dawley rats were divided into four groups (control, Doxorubicin 4 mg/kg treatment, Doxorubicin 4 mg/kg + 200 mg/kg extract, Doxorubicin 4 mg/kg + 400 mg/kg extract) and after 4 weeks, blood and cardiac tissues were analyzed. Animals treated with extract showed a reduction in cardiac damage biomarker LDH and creatine phosphokinase-MB (CK-MB) as well as the increase of mtDNA-CN and PGC-1α and Nrf2 expression. On the other hand, no modulation in SOD and 8-hydroxy-2'-deoxyguanosine (8-OH-dG) was detected (Patintingan et al. 2023).

### Ephedra sinica

*Ephedra sinica* is an Asian herbal extract traditionally used in the treatment of asthma, coughs and fever. It exerts several different biological effects such as anti-inflammatory, antioxidant and anti-obesity. Park and colleagues investigated the properties of *E. sinica* aqueous extract in mouse inguinal pre-adipocytes (miPA) and in human adipose-derived stem cells (hADSCs). Data showed that *E. sinica* extract was effective in inhibiting adipogenesis process in miPA by suppressing lipid accumulation and expression levels of adipogenic markers. Moreover, it was able to ameliorate mitochondrial function by inducing the expression of mitochondrial related genes and by increasing Nrf2 and mtDNA-CN at a concentration of 10 µg/mL in miPA differentiated cells (Park et al. 2022).

### Punica granatum

Pomegranate (*Punica granatum* L.) is considered a natural antioxidant substance rich in beneficial active compounds. The commercial dietary extract, standardized in ellagitannin content and generally recognized as safe (GRAS) by Food and Drug Administration (FDA), was investigated for its anti-proliferative properties in human oral cancer (Ca9—22, HSC-3, OC-2) cells. Peng and colleagues demonstrated that Pomegranate extract exerted anticancer effect through the induction of mitochondrial dysfunction in the tumor microenvironment. Involved mechanisms included depletion of ATP sources, disruption of mitochondrial membrane potential, trigger of mitochondrial SOD, reduction of mtDNA-CN and biogenesis both at 50 and 100 µg/mL (Peng et al. 2021).

## Oregonin from *Alnus incana*

Oregonin (1,7-bis-(3,4-dihydroxyphenyl)−3-hydroxyheptane-5-Ob-D-xylopyranoside) is a diarylheptanoid glycoside extracted from grey alder barks of *Alnus incana*. This molecule is already known for its ability to decrease lipid peroxidation markers (lipase, catalase, malondialdehyde levels), to enhance both GPx and SOD activity and to exert antioxidant, anti-inflammatory, antibacterial and antifungal properties. Krasilnikova and colleagues investigated for the first time Oregonin epigenetic regulation in cells, beside its ability to modulate DNA methylating enzyme expression and mtDNA-CN. Currently, increasing evidence shows how mitochondria play a key role in the epigenetic regulation of cells, by influencing cytosine methylation levels in the nucleus, which, in turn, can change based on the increase or decrease of mtDNA-CN. Experiments were conducted by treating mouse embryonic fibroblasts (MEFs), prepared from 13- to 14-day old embryos of mice and mouse embryo fibroblast cells (NIH/3T3). Cells treated with Oregonin 100 µM showed a significant increase of mtDNA-CN both in MEF and NIH/3T3 cell lines, with a trend dependent on the cell type, as the second cell model showed a higher genomic stability. On the other hand, although mtDNA-CN in MEF significantly increased at the dose of 100 µM, cell viability was significantly affected after 24 h of treatment, only. These data suggested a close relationship between mtDNA-CN amount and the expression of DNA methyltransferases family (Dnmts) mRNAs, even if additional studies are required in order to establish a non-cytotoxic dose (Krasilnikova et al. 2018).

## Apigenin

Apigenin is a naturally occurring flavonoid with a number of well-established biological properties including amelioration of muscle atrophy due to denervation, which age-related effects need to be elucidated. A study by Wang and colleagues, conducted in C57LB/6 male mice, randomly administered 25, 50 and 100 mg/kg/day of Apigenin (corresponding to low, moderate and high dose, respectively), for 9 months. Authors demonstrated how Apigenin was able to ameliorate age-related muscle atrophy in male mice in a dose-dependent manner. Moreover, it exerted an interesting antioxidant activity by inducing SOD, GPx, mitochondrial respiratory enzyme complexes I, II, IV, promoting ATP and factors involved in mitochondrial biogenesis. Apigenin also increased mitochondria size, number, volume and mtDNA-CN with statistically significant data at doses of 50 and 100 mg/kg/day versus control (Wang et al. 2020).

## Discussion

**Fig. 1:**
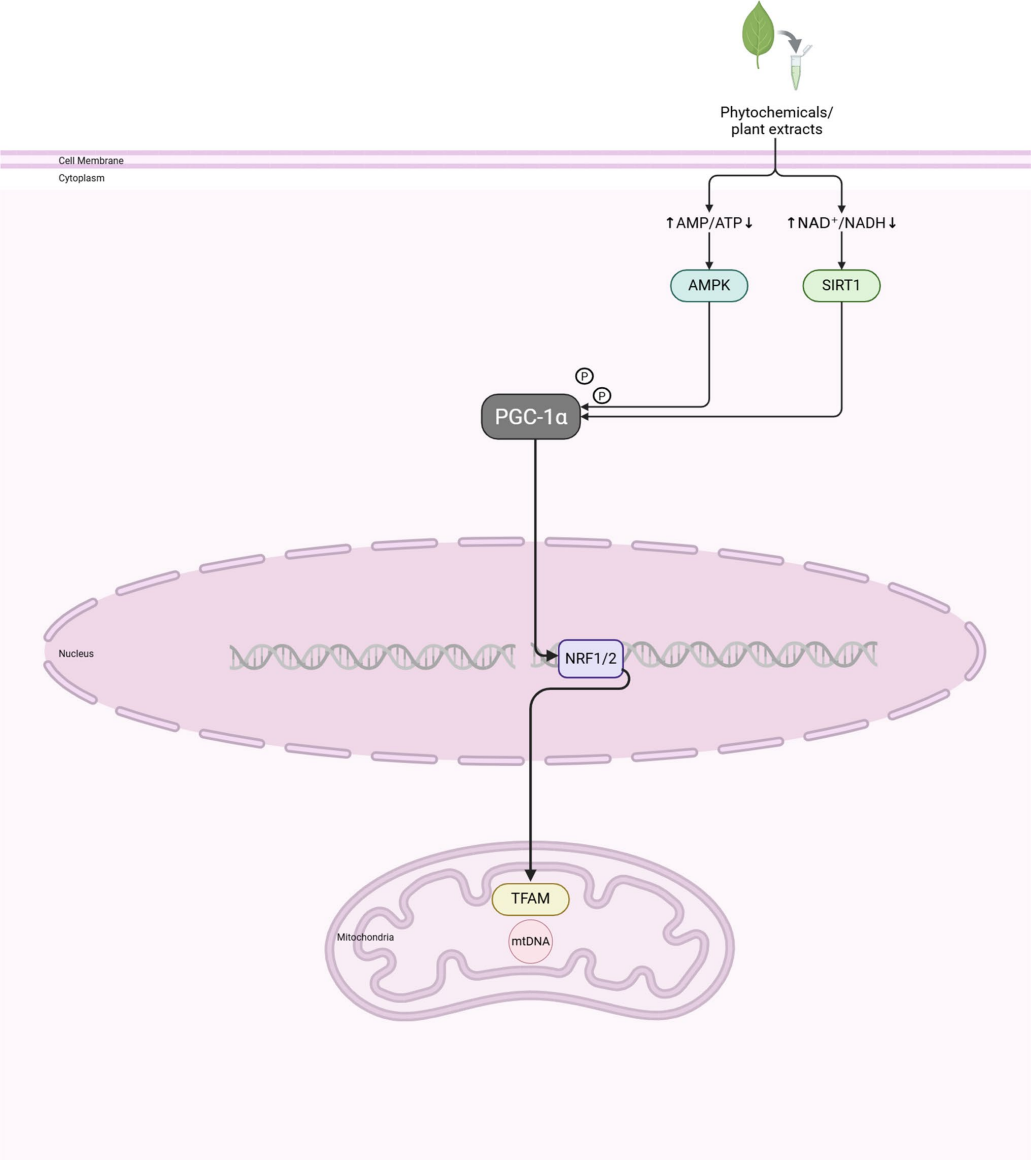
Phytochemicals and plant extracts affect mitochondrial biogenesis. Phytochemicals and plant extracts enhance mitochondrial biogenesis through the stimulation of AMP -activated protein kinase (AMPK) and Sirtuin 1 (SIRT1). The phosphorylation of peroxisome proliferator-activated receptor gamma co-activator (PGC-1α) regulates the activity of Nuclear Respiratory factor 1/2 (NRF-1/2), fostering the expression of Transcription Factor A, Mitochondrial (TFAM) and nuclear-encoded mitochondrial proteins

Plant extracts and phytochemicals obtained from a wide range of plants have shown potential in modulating mtDNA-CN through mechanisms that include the activation of mitochondrial biogenesis, antioxidant effects, and DNA repair. Many phytochemicals, especially Berberine, Resveratrol and EGCG, can naturally enhance mitochondrial biogenesis through the direct stimulation of Adenosine 5 ‘-monophosphate (AMP)-activated protein kinase (AMPK) and nicotinamide adenine dinucleotide (NAD^+^)-dependent deacetylase, which in turn activate SIRT1. This protein phosphorylates PGC-1α, the master regulator of mitochondrial biogenesis. Subsequently, PGC-1α binds peroxisome PPARγ and regulates NRF-1/2, fostering the expression of TFAM and nuclear-encoded mitochondrial proteins (Fig. 1). (Chen et al. 2023a).

Pomegranate extracts showed the ability to directly modulate mitochondrial homeostasis through the stimulation of Transcription factor EB (TFEB), a master regulator of mitophagy and lysosomal genes, whose activation is often linked to healthy lifespan and longevity (Lapierre et al. 2013)**.** The way by which pomegranate extracts trigger TFEB seems to be regulated by intracellular Ca^2+^ levels, rather than the known ERK1/2 and mTORC1 pathways, as MMP changes enhance the autophagosomes assembly (Tan et al. 2019).

**Fig. 2:**
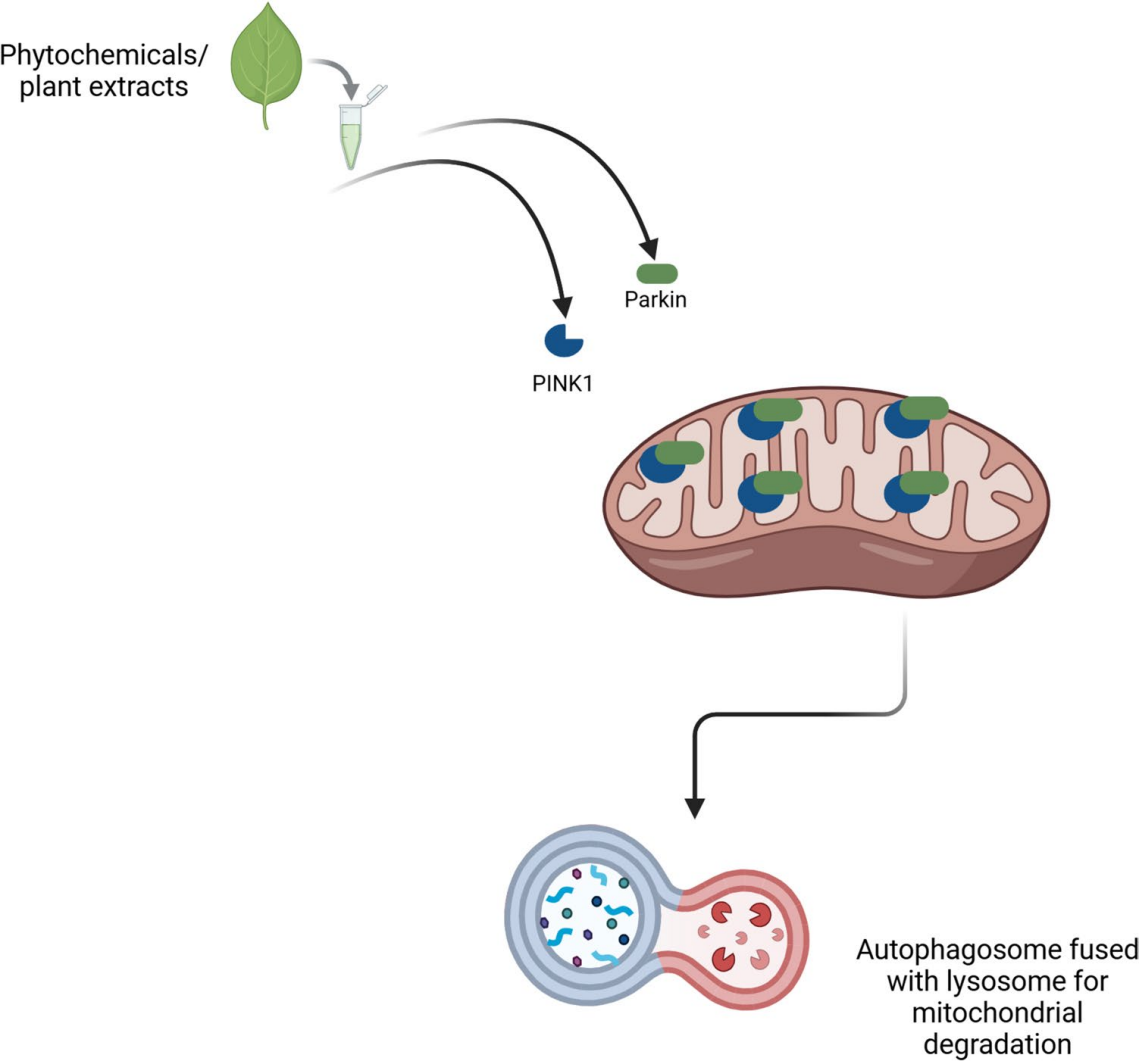
Phytochemicals and plant extracts improve mitophagy. Phytochemicals and plant extracts potentiate the recruitment of PTEN-induced kinase 1 (PINK 1) and Parkin on the Outer Mitochondrial Membrane (OMM) in response to mitochondrial damage, promoting mitophagy

Moreover, under stress conditions, pomegranate extracts potentiate the recruitment of PINK 1 and Parkin to the mitochondria, supporting an effective mitochondrial degradation and quality control to preserve mitochondrial health (Fig. 2) (Tan et al. 2019).

A growing body of evidence revealed that plant extracts and phytochemicals affect several mitochondrial pathways involved in the onset of neurodegeneration, metabolic disorders and cancers, highlighting their therapeutic potential in managing mitochondrial dysfunction and related diseases. Mitochondrial integrity is essential for the maintenance of the correct metabolic processes in the whole body. Indeed, impaired mitochondrial functions and reduced mitochondrial energetic activity are two common elements found in T2DM, thus suggesting a potential reduction of the enzymatic activity of OXPHOS complex I in patients affected by that condition (Li et al. 2016). In metabolic disorders, the administration of extracts and phytochemicals showed to increase insulin sensitivity, AMPK, PGC-1α receptor activity and SIRT1 signaling pathway, improving mitochondrial content and functions in several animal and cellular models (Baur et al. 2006; Li et al. 2016; Deng et al. 2019b). This positive aspect might be explained by the capability of bioactive molecules to block complex III of OXPHOS, phosphodiesterase or ATP synthase, thus contributing to the activation of AMPK cascade (Park et al. 2012).

**Fig. 3:**
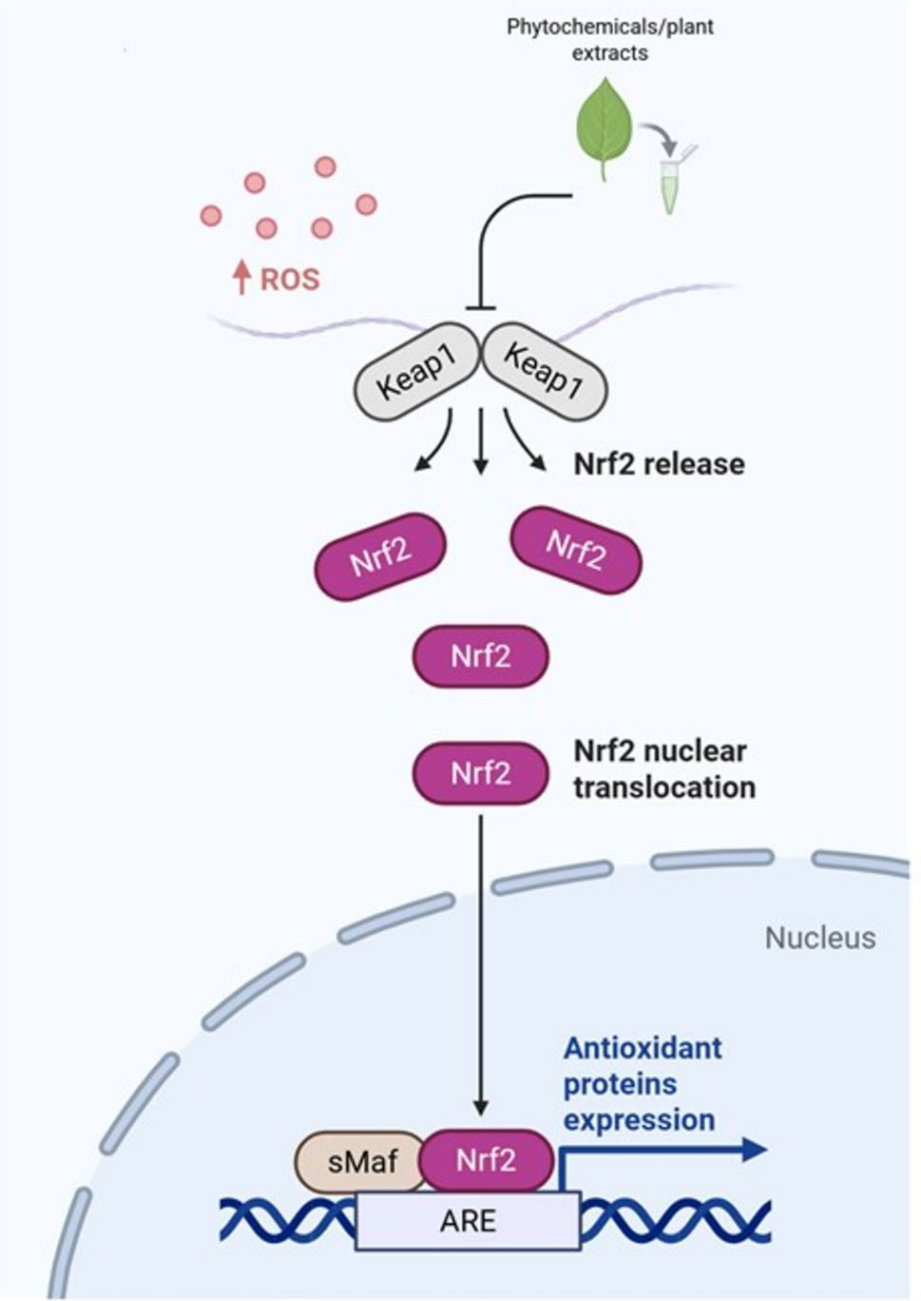
Phytochemicals and plant extracts counteract oxidative stress in condition of insulin resistance. Phytochemicals and plant extracts can reduce ROS levels by inhibiting the binding between Keap1 and Nrf2. In this way, the degradation of Nrf2 is prevented, and Nrf2 is translocated into the nucleus where it upregulates the antioxidant response element (ARE) pathway, thus facilitating the production of antioxidant enzymes

**Fig. 4:**
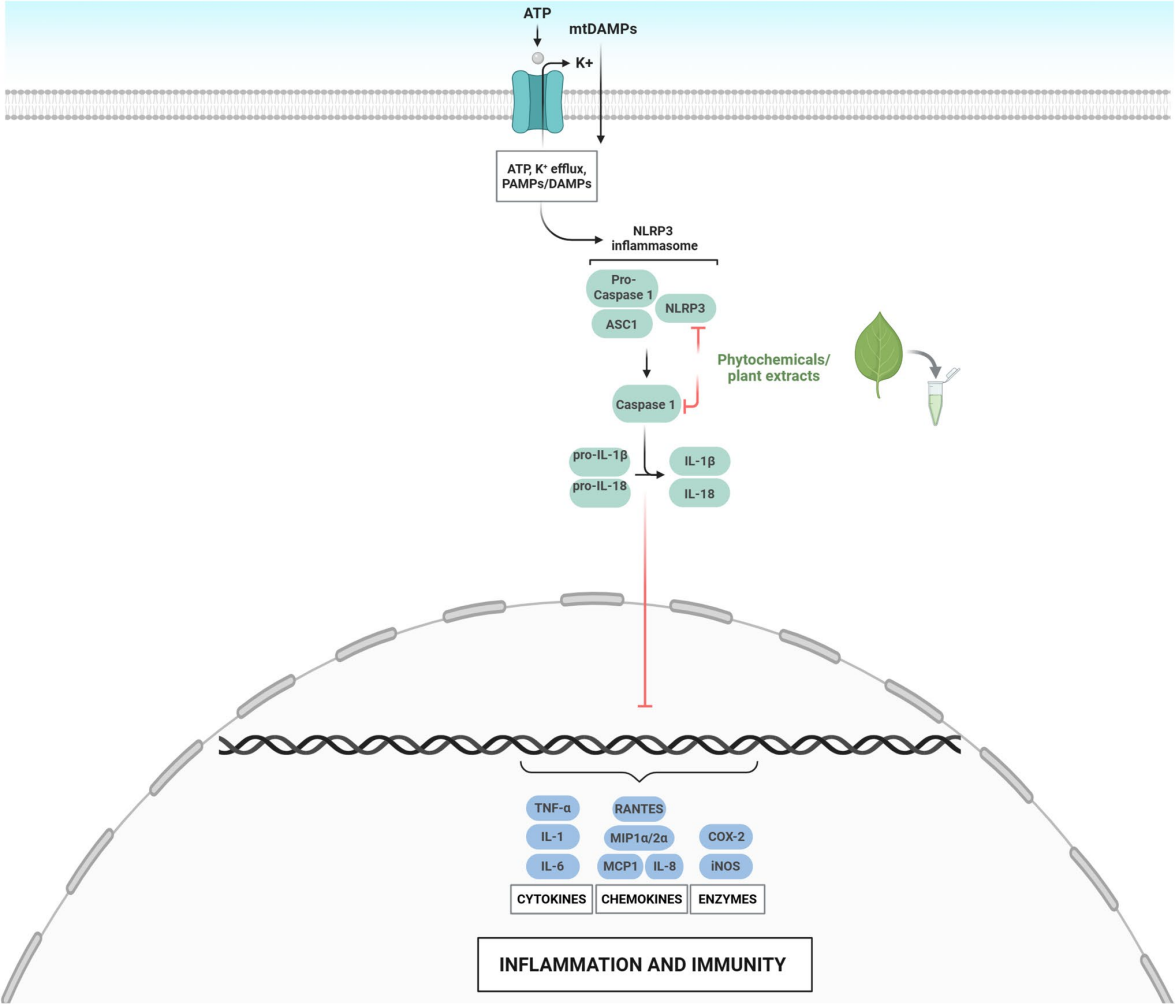
Phytochemicals and plant extracts ameliorate insulin secretion by preventing the activation of inflammosome. Phytochemicals and plant extracts could suppress the NLRP3 inflammosome assembly and the recruitment of Caspase 1, blocking the cleavage of pro- IL-1β, responsible for the activation of cytokines and chemokines involved in inflammation processes

Insulin resistance is a critical mechanism involved in the onset of T2DM, also exacerbated by oxidative stress and imbalance in redox homeostasis because of mitochondrial damage. Curcumin proved its efficacy in counteracting mitochondrial redox balance and ROS levels, enhancing the expression of Nrf2 in the muscle and liver tissue of diabetic mice. Specifically, curcumin promotes protein kinase C (PKC) phosphorylation and inhibits the binding between Keap1 and Nrf2 (Fig. 3). In this way, the ubiquitination and degradation of Nrf2 is prevented, as well as the expression of proinflammatory cytokines (Ren et al. 2019). Similarly, Ginsenoside Rg1 was shown to ameliorate insulin secretion, reducing the activity of NLRP3 and upregulating the Nrf2- antioxidant response element (ARE) pathway. This mechanism is able to restore mitochondrial function against oxidative stress, facilitating the production of antioxidant enzymes and thus maintaining ROS homeostasis (Fig. 3, Fig. 4) (Gao et al. 2020).

T2DM is also characterized by an alteration of mitophagic pathway which compromises mitochondrial quality control. Indeed, metabolic alterations exerted by T2DM promote depolarization of mitochondria and disruption of regular proteolytic processing of PTEN-induced kinase 1 (PINK1), consequently triggering the initiation of mitophagy. The administration of phenolic compounds in diabetic animal models showed to suppress PINK1 levels, thus demonstrating that these compounds actively participate to restore mitophagy and to ensure proper regulation of mitochondrial quality (Deshmukh et al. 2024). These results suggest that natural bioactive compounds, promoting mitochondrial biogenesis, could be considered a promising tool for the treatment of metabolic and obesity-related disorders.

The property of phytochemicals to modulate these pathways also represents a potential approach to cancer treatment, targeting molecules involved in cancer development and progression. These compounds show a direct anticancer effect, influencing the metabolism of cancer cells, inhibiting cells growth, and disrupting crucial metabolic processes, such as carbohydrate metabolism, lipid metabolism, and protein synthesis (Kooshki et al. 2022).

**Fig. 5:**
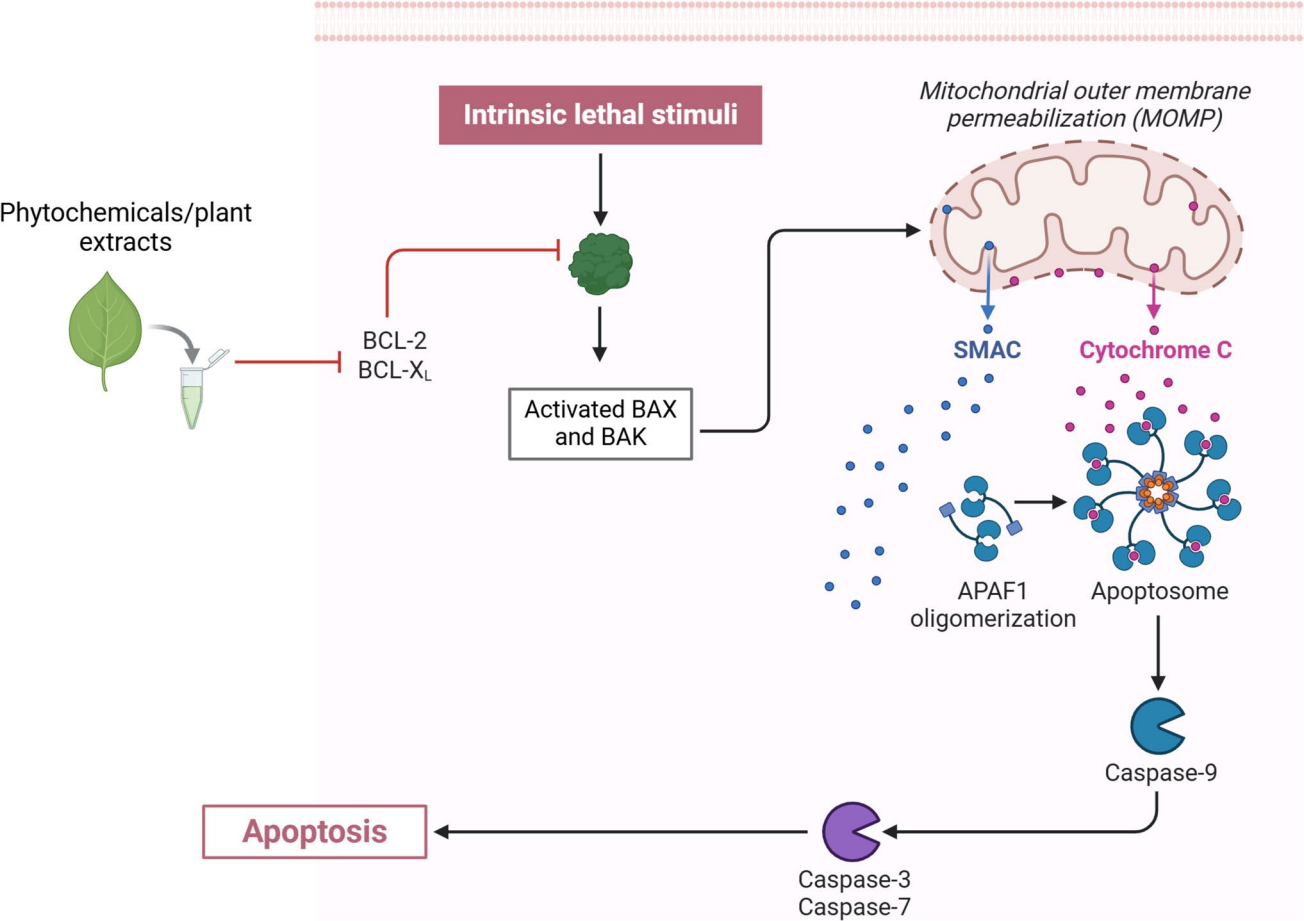
Phytochemicals and plant extracts promote apoptosis of cancer cells. Phytochemicals and plant extracts are able to reduce the expression of Bcl-2/Bcl-XL anti-apoptotic proteins. The further activation of BAX and BAK increases the permeabilization of the Outer Mitochondrial Membrane (OMM), promoting the activation of caspase-dependent apoptotic pathway

Cancer cells frequently increase their resistance to chemotherapy treatments through a mechanism known as mitochondrial drug resistance, where the overexpression of anti-apoptotic proteins inhibit the release of cytochrome c from the mitochondria (Sitarek et al. 2022). The administration of Lycopene strongly reduced the expression of Bcl-2 protein, enhanced mitochondrial permeability and activated caspase-dependent apoptotic pathway in both prostate cancer and cervical cancer cells (Fig. 5) (Das S. 2023).

Another compound which revealed its anticancer property, inducing apoptosis via mitochondrial pathway, is Halimane, isolated from *Plectranthus ornatus*. This phytochemical demonstrated to induce apoptosis via mitochondrial accumulation of ROS, promoting mitochondrial damage by altering mitochondrial membrane potential. Moreover, its action was also able to modify mtDNA-CN levels and to increase mitochondrial DNA damage in the *ND1* and *ND5* gene regions in human breast adenocarcinoma and human head and neck squamous cell lines (Sitarek et al. 2022).

The capability of phytochemicals to restore mitochondrial activity and mtDNA-CN levels, could also reflect their property to modulate ROS levels in cancer cells, increasing their sensitivity to chemotherapeutic drugs and opening new fields for a better management of therapeutical interventions (Sitarek et al. 2022). The identification of bioactive substances with the property to counteract oxidative damage and to reestablish mitochondrial dysfunction might represent a successful approach against the progression of neurodegenerative disorders.

Stress stimuli and altered cellular metabolism might trigger an increase in ROS production and a depletion of antioxidant defense systems, resulting in a defective mitochondrial respiratory chain, mutation in mtDNA, and altered Ca^2+^ homeostasis. These events could promote apoptosis, leading the aging process through the progression towards cognitive decline and disease development (Ullah et al. 2021). The neuroprotective action exerted by several metabolites encompasses the inhibition of pathogenic protein aggregates accumulation, supporting the proteolytic system to mitigate neuronal dysfunction (Uddin et al. 2020). Studies performed in animal models demonstrated that phytochemicals can mitigate mitochondrial dysfunctions in neurodegenerative disorders through the modulation of AMPK- PGC-1α pathway, improving both mitochondrial activity and content, and restoring Cyclooxygenase (COX) activity and ATP production. These results proved that the use of these natural compounds might enhance mitochondrial functions and dynamics, thus exerting protective effect against neuronal loss and synaptic plasticity (Yan et al. 2025).

Oxidative stress, resulting from an excessive production of ROS species, has been recognized as a key factor in the promotion of brain aging, accumulation of Aβ peptides deposits, and neurodegeneration. It has been proposed that Resveratrol actively modulated mitochondrial functions through the activation of phosphatidylinositol-3-kinase (PI3K)/Akt/Nrf2 pathways, inhibiting tau protein hyperphosphorylation and increasing the viability in Aβ-treated PC12 cells. This mechanism proved to prevent the overexpression of mitochondrial structural genes, thus preserving mitochondrial functions and protecting cells against Aβ toxicity (Manczak et al. 2010).

EGCG showed neuroprotective effects in PD pathogenesis. Indeed, the administration of this compound improved electron transport chain (ETC) efficiency, preserving the inhibition of complex I and rescuing ETC from electron leakage. EGCG was also able to counteract cells death, removing damaged mitochondria and triggering mitophagy through the direct activation of PINK1/Parkin pathway (Kamboj et al. 2025). Several dietary phytochemicals, derived from plant-based foods, could act as powerful scavenging free radicals, modulating oxidative stress and preventing cellular damage linked to chronic inflammatory diseases (Pandey et al. 2009; Pierzchalska et al. 2016; Hajam et al. 2022).

Despite promising preclinical results, further clinical studies are essential to confirm the effectiveness of these plant-based interventions in humans. Additionally, the exact molecular pathways and optimal dosages need to be better understood to translate these findings into practical therapeutic applications.

Results coming from clinical trials which investigated Resveratrol’s health benefits are still controversial, maybe because of the heterogeneity of the enrolled patients or for the hormetic property of this compound which limits its usage (Calabrese et al. 2010; Tome-Carneiro et al. 2013). The major limitation of Resveratrol lies in its low bioavailability and solubility, but also in the further modifications that happen in the liver, where Resveratrol is converted in methylated products which reduce the power of its biological activity (Walle et al. 2004).

Curcumin seems to share the same limitations of Resveratrol, since its poor absorption and its rapid metabolism represent a serious challenge despite its anti-oxidant and anti-inflammatory properties (Sohn et al. 2021).

Metabolites of Resveratrol, such as o-Quinone, promote the inhibition of P450 oxidative enzymes and are often associated with toxic effects and induction of oxidative stress (Bolton et al. 2018). Moreover, it has been demonstrated that pro-oxidant effect of Resveratrol and Curcumin could promote DNA damage and the activation of pro-apoptotic pathways (de la Lastra et al. 2007).

Indeed, Curcumin caused DNA damage to the mitochondrial and nuclear genomes in HepG2 human hepatocellular carcinoma cells, maybe through the direct alteration of topoisomerase II (Cao et al. 2006).

Higher doses of Curcumin also generated an increase in ROS species, altering the activity of thioredoxin reductase, a key factor in the promotion of carcinogenesis (Gupta et al. 2020). Moreover, Curcumin showed angiogenesis properties, as an increase in Vascular Endothelial Growth Factor (VEGF) expression has been observed after the administration of this compound (Gururaj et al. 2002).

Nevertheless, Curcumin exhibited a greater uptake by cancer cells compared to normal cells, as well as the pro-oxidant action of Resveratrol has revealed its efficacy as chemotherapeutic agents against melanoma cells and ovarian cancer cells, where high concentration of this compound lead to cell cycle arrest and caspase- dependent cell death (Kunwar et al. 2008; Heo et al. 2018; Kim et al. 2019).

Recent evidence suggested that the combination of Resveratrol with other bioactive molecules could enhance its therapeutical effects. The inclusion of Resveratrol into poly-lactic-co-glycolic acid (PLGA) nanoparticles has been shown to increase its oral bioavailability in rats (Siu et al. 2018).

Curcumin liposomes formulated with phospholipids, cholesterol, and Tween-80 have been shown to enhance its stability across diverse pH levels, improving the absorption and the antioxidant capabilities of this compound (Tai et al. 2020).

Furthermore, a combination of Curcumin, Resveratrol and Epicatechin gallate inhibited tumor growth in human papillomavirus (HPV)-positive head and neck squamous cell carcinoma, limiting tumor spheres formation and increasing p53 protein levels (Mukherjee et al. 2017)**.**

Despite these limitations, phytochemicals and plant extracts have been elected as promising to both mitigate and restore diseases and dysregulated pathways. The chance to use natural molecules, alone or in combination, has the advantage of limiting the adverse side effects commonly related to traditional drug treatments, as frequently observed in cancer. To the same extent, these substances might represent a valuable alternative to prevent or treat aging-related and neurodegenerative disorders, for which effective therapeutical approaches are still challenging.

Pre-clinical evidences showed that plant extracts and phytochemicals represent a promising tool able to improve mitochondrial dynamics and bioenergetics and to prevent or treat several different mitochondrial-associated diseases through the molecular mechanisms and the involvement of signaling pathways largely discussed in this review. Although several steps forward have been made in order to translate these results into clinical practice, currently there is a gap between preclinical research and clinical trials that need to be filled. Emerging perspectives regarding innovative therapeutic strategies able to modulate mitochondrial health include the integration of synthetic, semi-synthetic and plant-derived compounds, an approach targeted to maximize beneficial properties and minimize side effects. Before transferring the use of plant-derived extracts or compounds to the clinic, it would be important to understand the potential side effects connected with their use, alone or in synergism, and interaction with other compounds. Another important factor that needs elucidation concerns the long-term effects of plant-derived extracts and compounds accumulation for mitochondrial health and the potential issue of exposure to hypersensitivity or allergic reactions. Furthermore, the use of compounds that need extraction, preparation and purification requires standardized methods able to guarantee same concentrations, bioactivity, efficacy and safety (Anchimowicz et al. 2025). All these findings are the limiting rate to clinical approach, even if, as previously discussed, two clinical trials were already conducted regarding the use of Rg in men with metabolic syndrome and KRG in post-menopausal women (Table 1).

Like many other substances, phytochemicals are absorbed and subjected to metabolism processes. These processes occur in the mouth, stomach, intestine, and the absorption phenomena are mediated by lymphatic circulation or blood through intestinal epithelial cells. On the other hand, non-absorbable substances reach the colon and are broken down by bacteria. In order to predict and simulate the journey of phytochemicals in the human body, Huang et al. (2021b), reviewed the in vitro simulation models of the natural phytochemicals absorption and metabolism. Currently, poor oral bioavailability and inefficient intestinal absorption represent a deterrent for research and development of new plant-derived active molecules (Huang et al. 2021b). Absorption, metabolism and distribution of phytochemicals such as psoralen, lignans, flavonoids like naringenin and hesperitin are influenced by genetic variation in the pathways: this means that circulating levels of these molecules present a very high variability among individuals. Glycosides molecules or conjugates need hydrolysis process, carried out by bacterial β-glucosidases (lactase phlorizin hydrolase) or by gut bacterial β-glucosidases colonizing small intestine and colon in order to be absorbed. Furthermore, hydrolyzed aglycones are subjected to first-pass metabolism in the liver, like most of the xenobiotics, and are conjugated with glutathione, glucuronic acid or sulfate (Lampe and Change 2007). Several of the best-known phytochemicals, including Ellagic Acid, Caffeic Acid, Chlorogenic Acid, but also Resveratrol and Curcumin, investigated in this review, are metabolized in the biological system. Metabolism, occurring in liver, gut, intestine, spleen and mediated by cytochrome P450 enzymes, is responsible for bioavailability, pharmacokinetics and effectiveness of phytochemicals. For example, metabolism of tea polyphenols in the human body was investigated after administration of 500 mL of green tea. After 24 h, urine and plasma were collected and analyzed: as a result, the major found secondary metabolites in blood were conjugates such as (-)-epicatechin-3’-O-glucuronide, (-)-epicatechin-3’-O-sulfate, 3’-O-methyl-(-)-epicatechin-O-sulfate. Another factor that need to be taken into account for drug metabolism is chirality: for example, (+)-catechin is more easily absorbed than (-)-catechin. Ellagitannins and Ellagic Acid are conversed to Urolithin A and its glucuronide conjugate thanks to the action of bacteria. This metabolite showed high anti-carcinogenic and anti-inflammatory properties and high bioavailability (Dixit et al. 2021). The structural changes that phytochemicals undergo during these operations sometimes translate into different biological activities. Berberine, a phytochemical discussed in this review, undergoes an extensive metabolism process after oral administration which lead to demethylation, demethylenation, reduction, hydroxylation and conjugation, in vivo. Produced active metabolites, such as Columbamine, Berberrubine, Demethyleneberberine, showed comparable pharmacological effects with Berberine (Wang et al. 2017).

Wang et al. (2012) reviewed both in vitro and in vivo reports concerning Lycopene and Lycopene derivatives, concluding that the biological activities, especially in chronic disease management, can be partly attributable to Lycopene metabolites (Wang et al. 2012). Not all phytochemical metabolites are always connected to more favorable bioavailability or enhanced biological activities: studies in this direction are yet inconclusive, needing further investigation (Ross et al. 2002). The limits of phytochemicals are related to their instability, low solubility/selectivity, poor bioavailability and chemical degradation. Sometimes, natural bioactive compounds are barely absorbed by the body, thus diminishing their efficacy when they reach the targeted tissues. Furthermore, the lack of well-designed clinical trials could lead to an underestimation of potential off-target effects and interactions with other medications.

Nowadays, new progress in the field of nutraceuticals and pharmaceuticals has been achieved, allowing the encapsulation of phytochemicals into nanoparticle-based carriers. This new delivery system ensures a gradual release of natural compounds, facilitating the absorption and efficacy and showing new opportunities for personalized supplementation strategies (Lu et al. 2021). Currently, different approaches and formulations aimed at improving parameters such as solubility, stability and bioavailability of plant extracts and phytochemicals are under investigation. Recently, solid dispersion gained attention and is considered a good strategy to overcome the solubility problem for hydrophobic compounds and thus enhance their oral bioavailability (Mohapatra et al. 2021).

Nanotechnology-based carriers are useful to enhance solubility, stability, bioavailability, target selectivity and overall pharmacokinetics parameters of phytochemicals delivery. The strategy consists in increasing the surface area by decreasing particle size and allowing the absorption of surfactants on phytochemical particles in order to promote micelles formation. Micelles are soluble in water, allowing phytochemicals to cross biological barriers (Teli et al. 2024). Phytochemicals also address problems connected with deterioration at warmth or light that can be partially resolved by research and development of lipid-based delivery technologies including liposomes and nanoemulsion. (Mehan et al. 2022).

## Conclusions

This review emphasizes the concrete beneficial effect of phytochemicals and plant extracts in ameliorating mitochondrial activity and health.

It has been proposed that plant secondary metabolites are able to ameliorate mitochondrial functions, activating key signaling pathways and transcriptional co-activators involved in mitochondrial biogenesis. Moreover, the administration of natural compounds has been shown to positively regulate mitochondrial dynamics, raising the expression of proteins involved in fusion processes, essential for the production of healthy mitochondria. Phytochemical compounds and plant-derived extracts such as *Ginkgo biloba* and *Panax ginseng* show potential in modulating mtDNA-CN and improving mitochondrial function. Bioactive compounds are naturally present in nature and highly tolerated by the human body, fostering their employment through diet or as food supplements.

Oxidative stress, often attributed to environmental and lifestyle changes, is a critical condition caused by an excessive production of ROS and nitrogen species which causes damages to biomolecules such as DNA, proteins, and lipids. This phenomenon is able to disrupt gene expression levels, negatively impacting health conditions. Plant extracts and their plethora of secondary metabolites, have shown a considerable property to counteract toxic oxygen species, thus limiting the side effect of oxidative stress and its adverse outcomes.

In this scenario, plant extracts and their bioactive compounds might act as powerful nutraceuticals through a multi-targeting approach, positively affecting multiple hallmarks of aging and diseases.

The discussed use of these compounds offers promising therapeutic avenues for disorders characterized by mitochondrial dysfunction, including neurodegenerative diseases, metabolic disorders, and aging-related conditions. Survey of the literature highlighted the lack of clinical studies concerning the modulation of mtDNA-CN by phytochemicals and plant extracts, and this can be partially attributed to the difficulty to set up well-structured and well-organized clinical studies in this direction. This work should represent the starting point to fill this gap.

However, further research needs to be established in order to test the clinical efficacy and safety of phytochemicals and plant extracts.

## Data Availability

Not Applicable, as no dataset were generated or analyzed in this paper.
